# Supplementary material for: Patient propagules: Do soil archives preserve the legacy of fungal and prokaryotic communities?
Source: PLoS One. 2020 Aug 11;15(8):e0237368. doi: 10.1371/journal.pone.0237368 (PMC7418970; doi:10.1371/journal.pone.0237368)
Supplement: S1 File — (PDF) [file pone.0237368.s001.pdf]

S1 Fig.

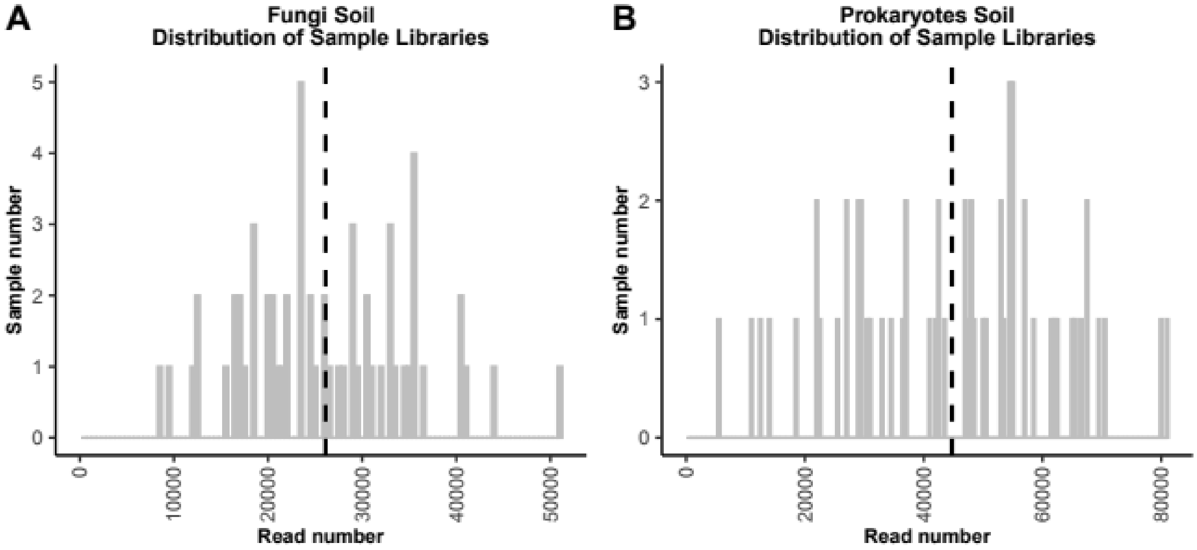

Distribution of sample libraries. (A) fungal and (B) prokaryotic communities.

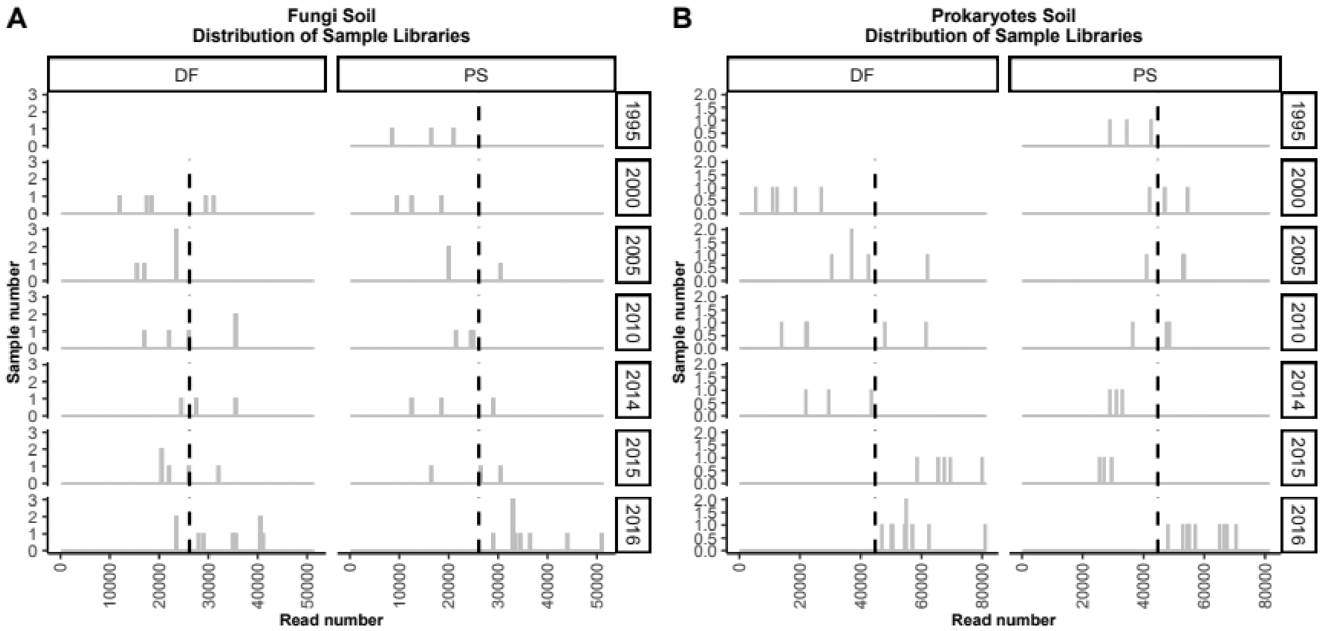

S2 Fig. Distribution of sample libraries separated by time. (A) fungal and (B) prokaryotic communities.

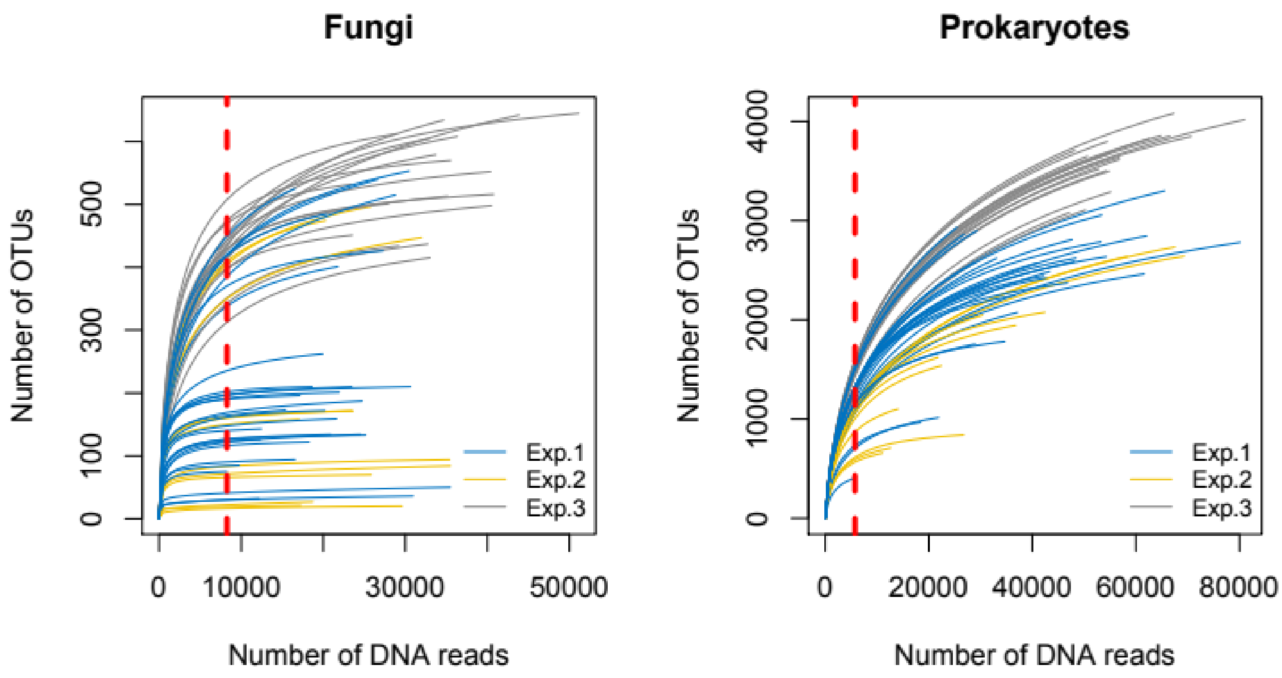

S3 Fig. Rarefaction curves for both fungal and bacterial samples.

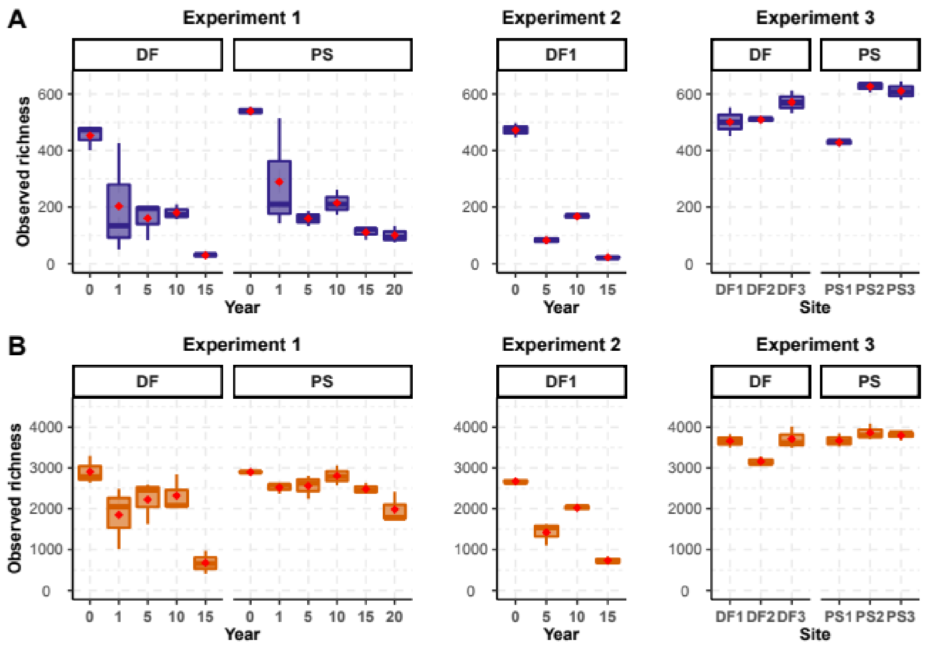

S4 Fig. Observed richnes boxplots. Fungal (A) and prokaryotic (B) observed richness boxplots (n=3) in Experiment 1, Experiment 2, and Experiment 3 (See M&M for details). Red diamonds represent the mean of the sample distribution.

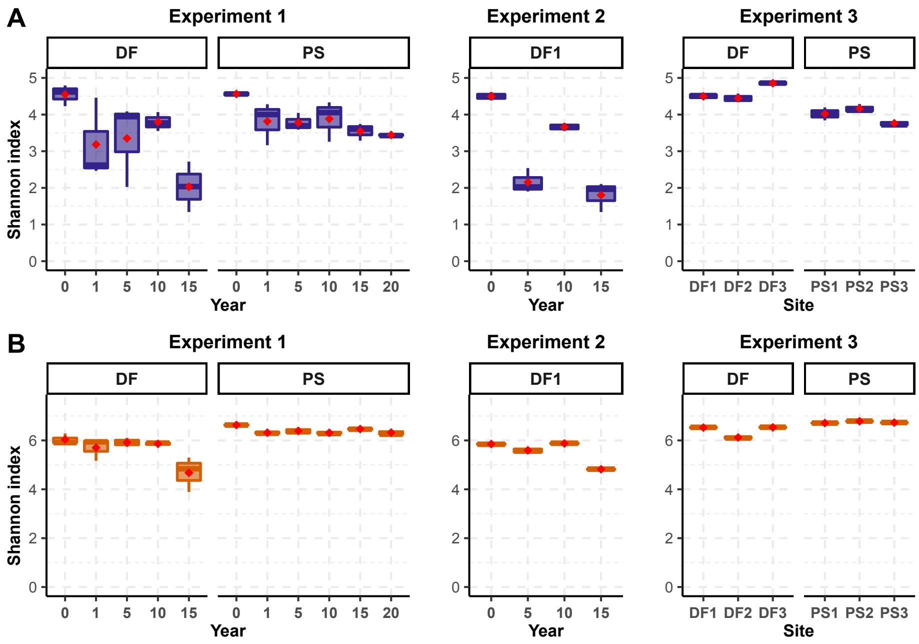

S5 Fig. Shannon index boxplots. Fungal (A) and prokaryotic (B) Shannon index boxplots (n=3) in Experiment 1, Experiment 2, and Experiment 3 (See M&M for details). Red diamonds represent the mean of the sample distribution.

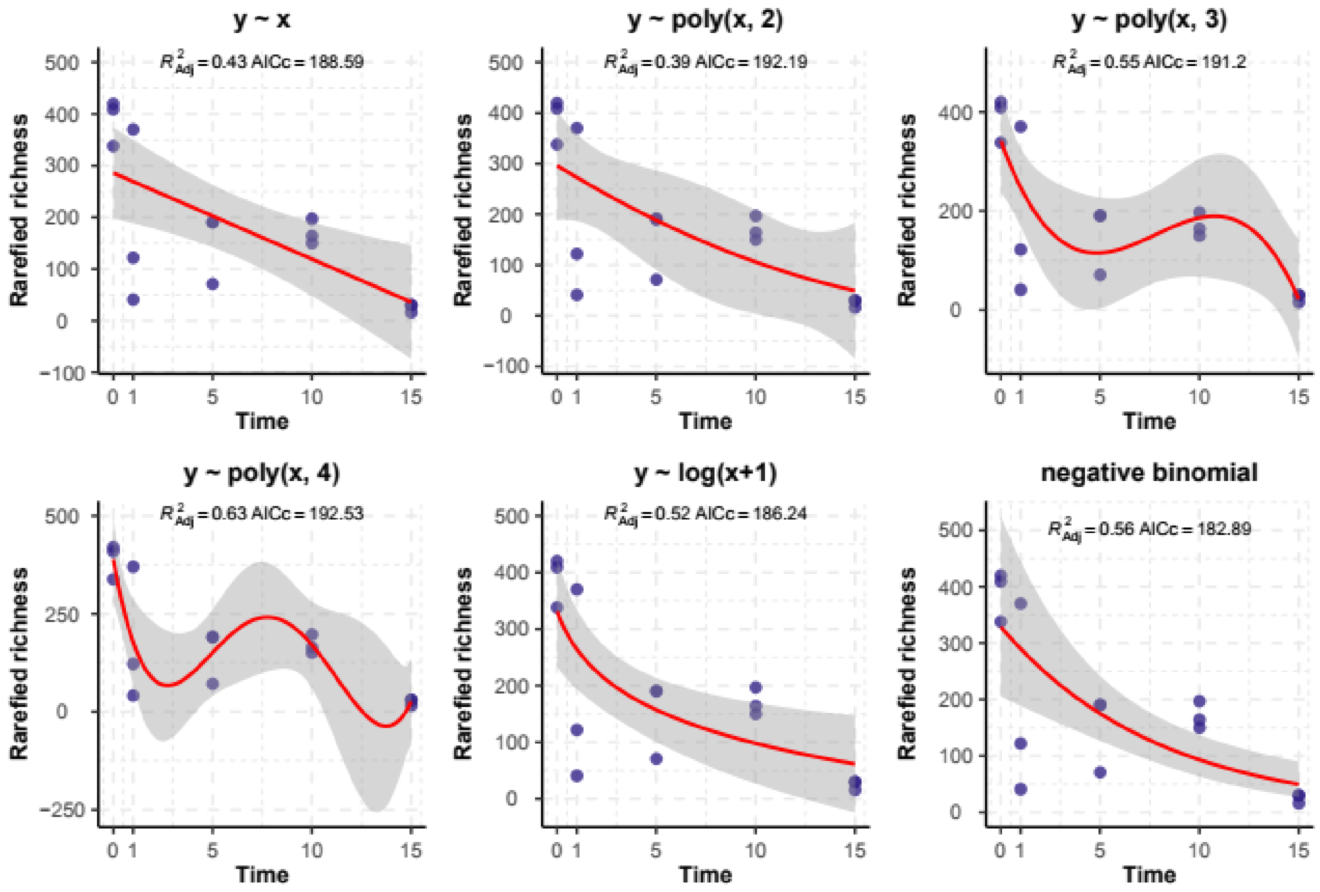

S6 Fig. Changes in fungal species richness over time for DF (deciduous forest) soils in Exp1. Different models to describe rarefied richness variation with increasing storage time. Adjusted R^2 and AICc are reported.

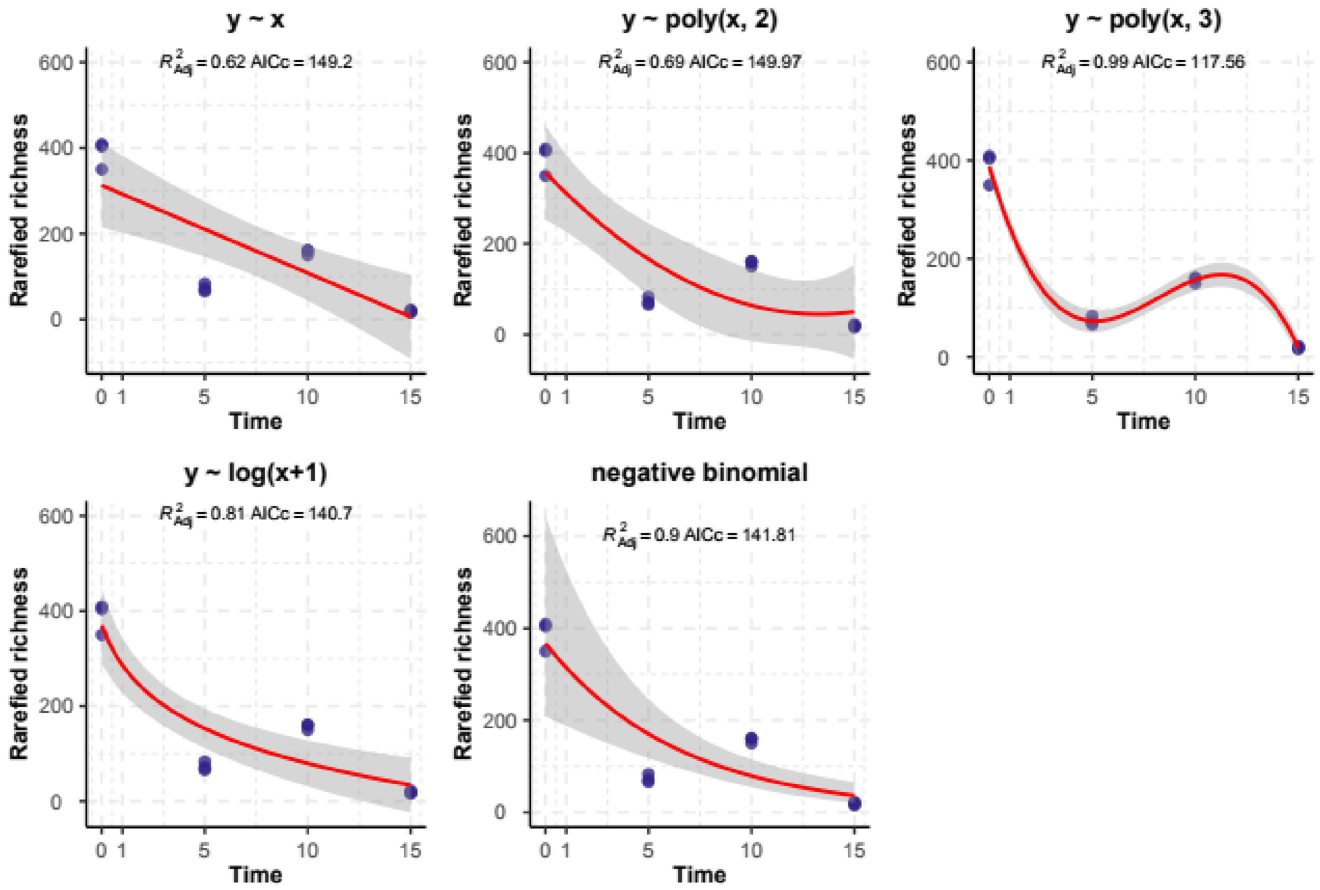

S7 Fig. Changes in fungal species richness over time for DF (deciduous forest) soils in Exp2. Different models to describe rarefied richness variation with increasing storage time. Adjusted R^2 and AICc are reported.

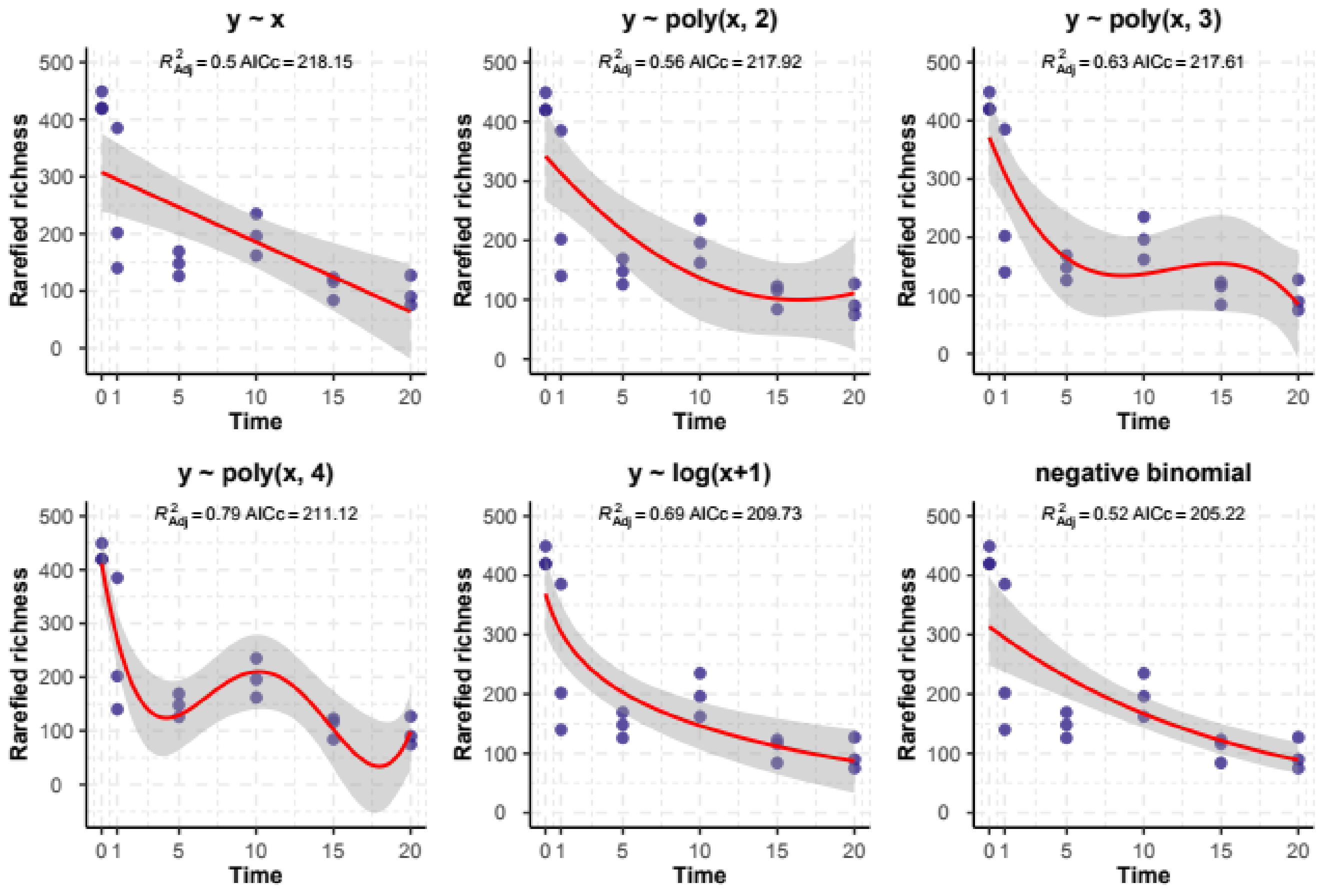

S8 Fig. Changes in fungal species richness over time for PS (*Populus* stand) soils in Exp1. Different models to describe rarefied richness variation with increasing storage time. Adjusted R^2 and AICc are reported.

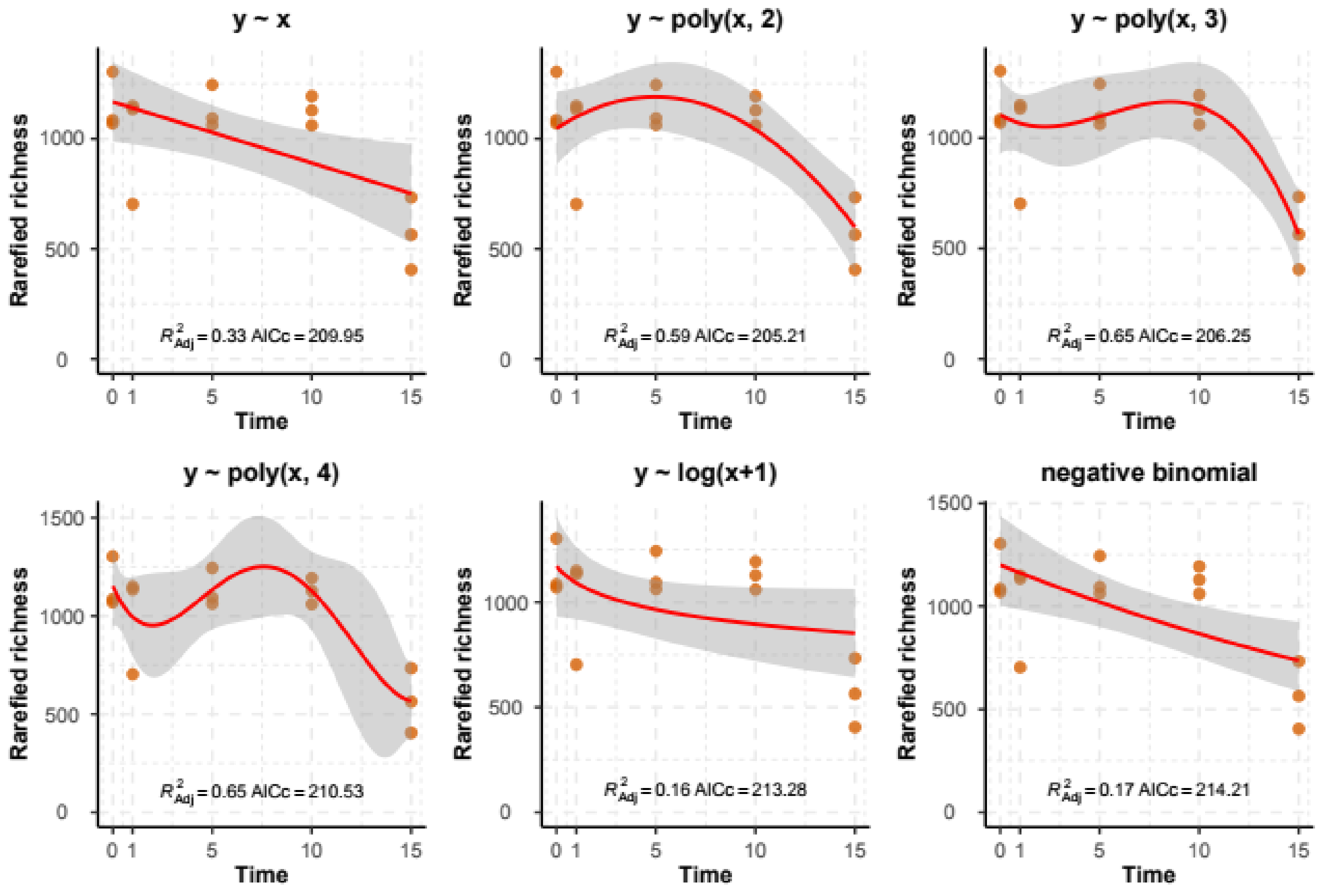

S9 Fig. Changes in prokaryotic species richness over time for DF (deciduous forest) soils in Exp1. Different models to describe rarefied richness variation with increasing storage time. Adjusted R^2 and AICc are reported.

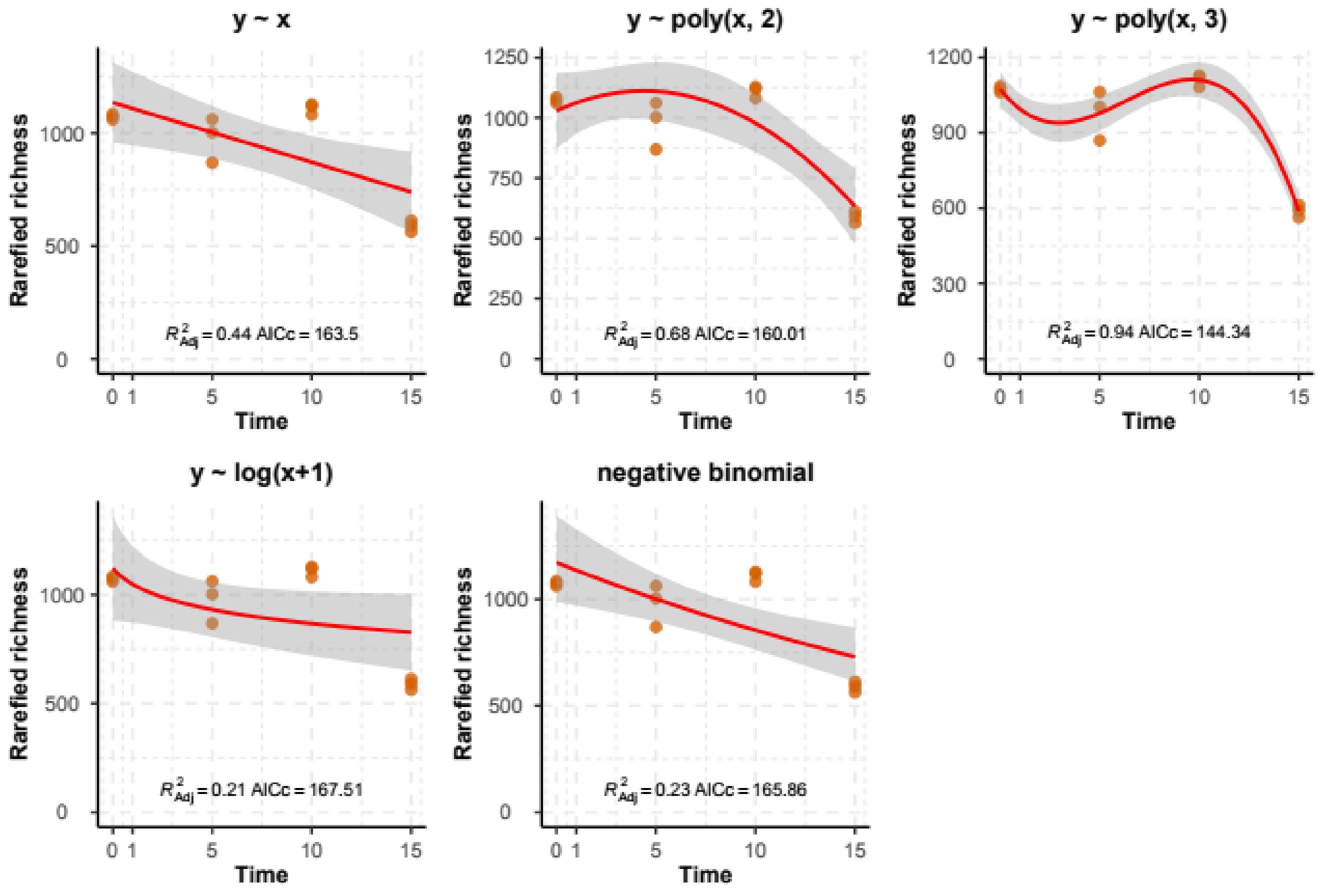

S10 Fig. Changes in prokaryotic species richness over time for DF (deciduous forest) soils in Exp2. Different models to describe rarefied richness variation with increasing storage time. Adjusted R^2 and AICc are reported.

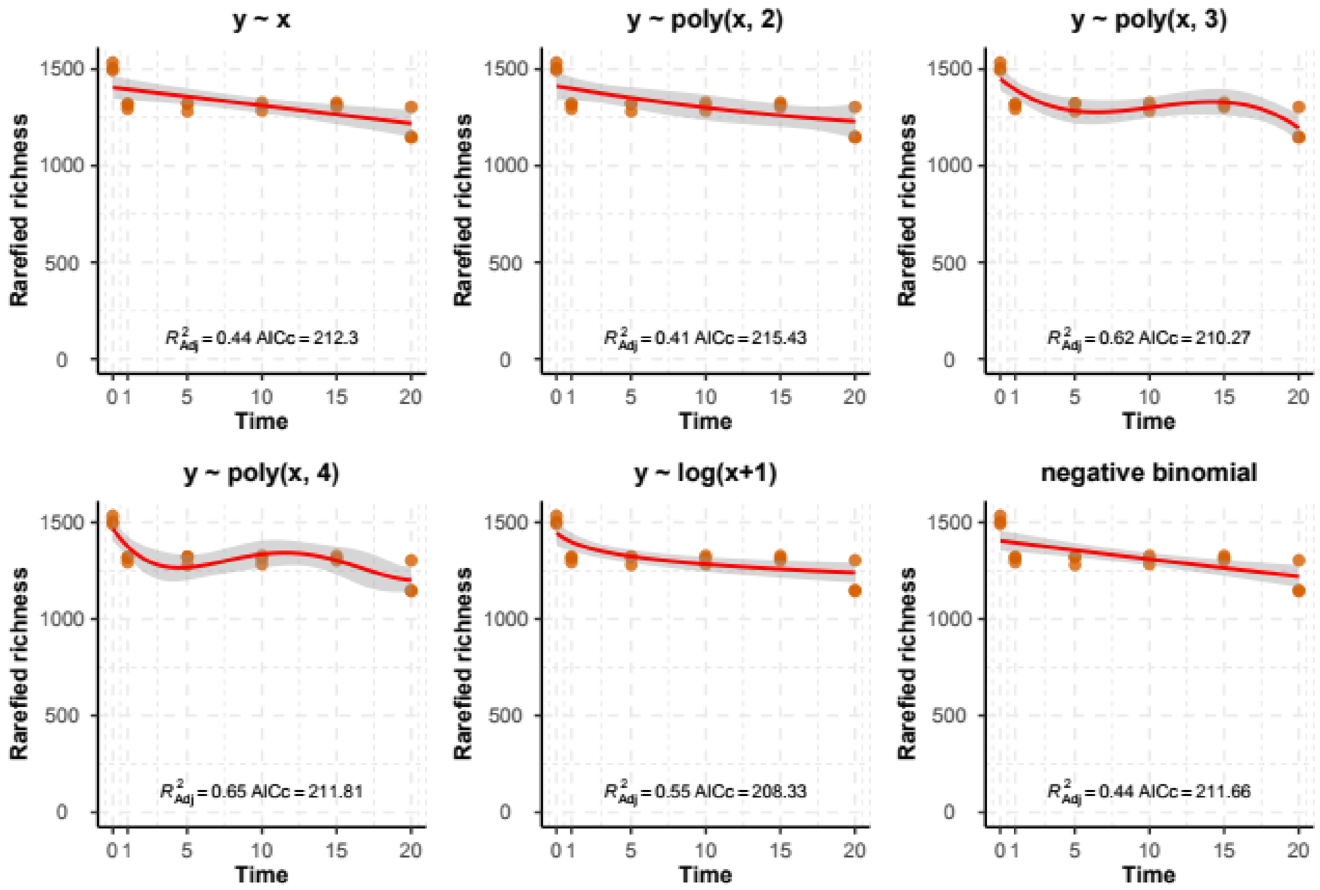

S11 Fig. Changes in prokaryotic species richness over time for PS (*Populus* stand) soils in Exp1. Different models to describe rarefied richness variation with increasing storage time. Adjusted R^2 and AICc are reported.

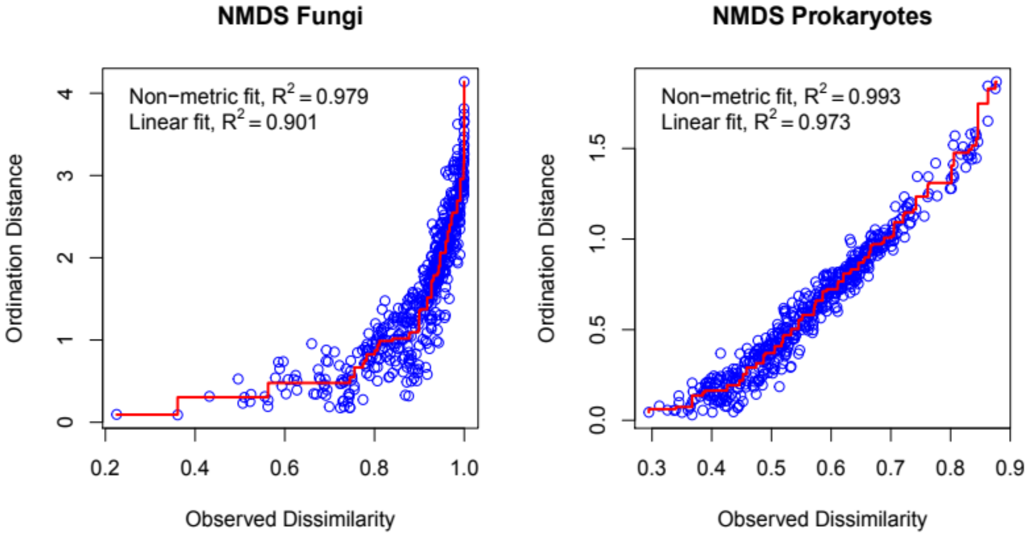

S12 Fig. Shepard stress-plot diagrams for ITS and 16S NMDS graphs reported in Fig 3. The plot shows ordinations distances against original dissimilarities. Non-metric fit is based on stress value of the NMDS ordination and calculated as R^2= *1-S*S*. Linear fit is the squared correlation between fitted values and ordination distances.

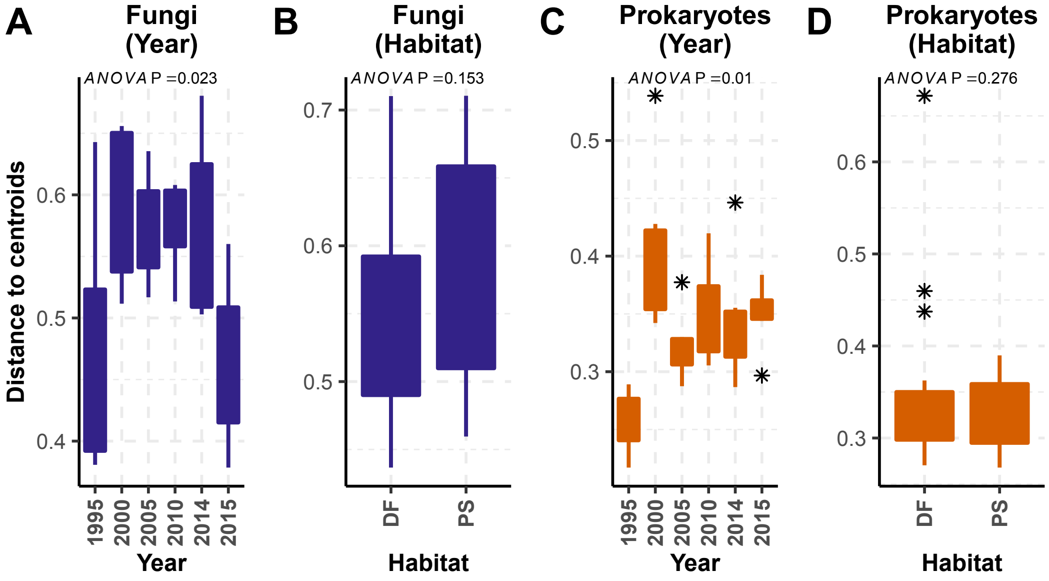

S13 Fig.

Boxplots of Distance from centroids to assess for homogeneity of group variances (function “betadisper” in *vegan* R package). (A) fungi year, (B) fungi habiatat, (C) prokaryotes year, and (D) prokaryotes habiatat. Permutational ANOVA (perm. 999) was used to assess significant differences at p=0.05.

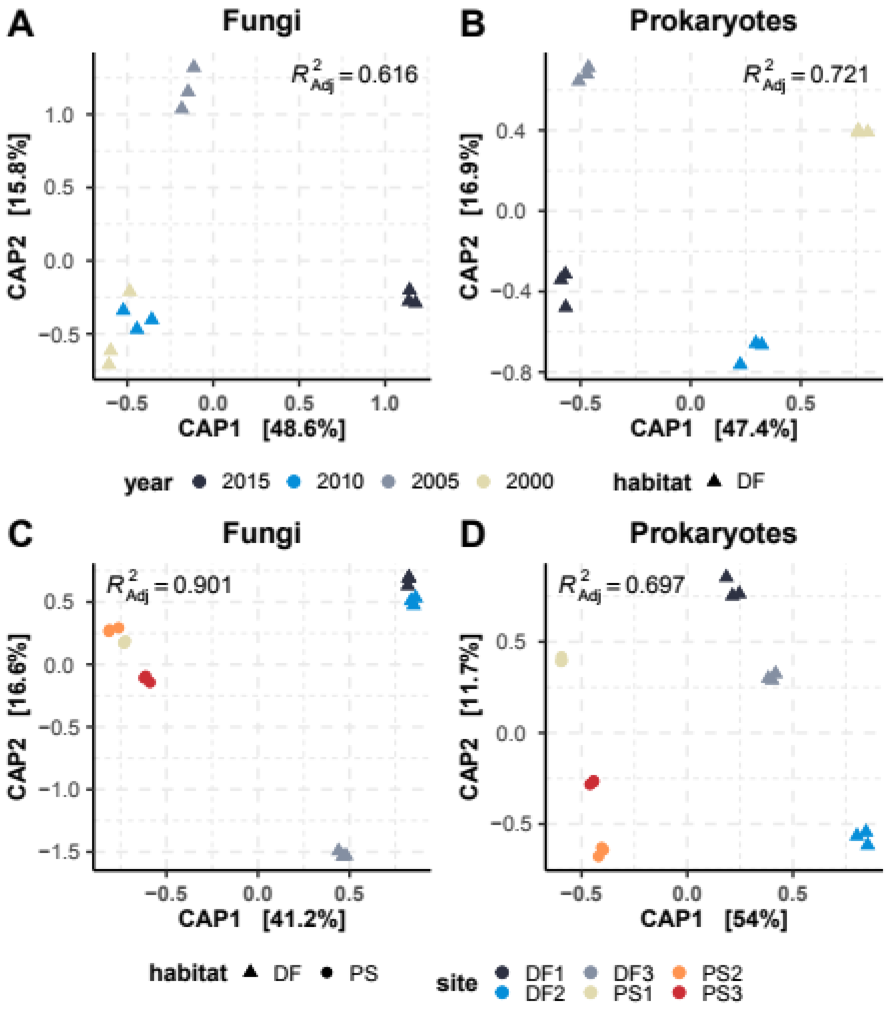

S14 Fig. Canonical analysis of principal coordinates (CAP) constrained ordinations. Exp2 fungal (A), Exp2 prokaryotic (B), Exp 3 for fungal (C) and Exp3 prokaryotic (D) communities ordination graphs.

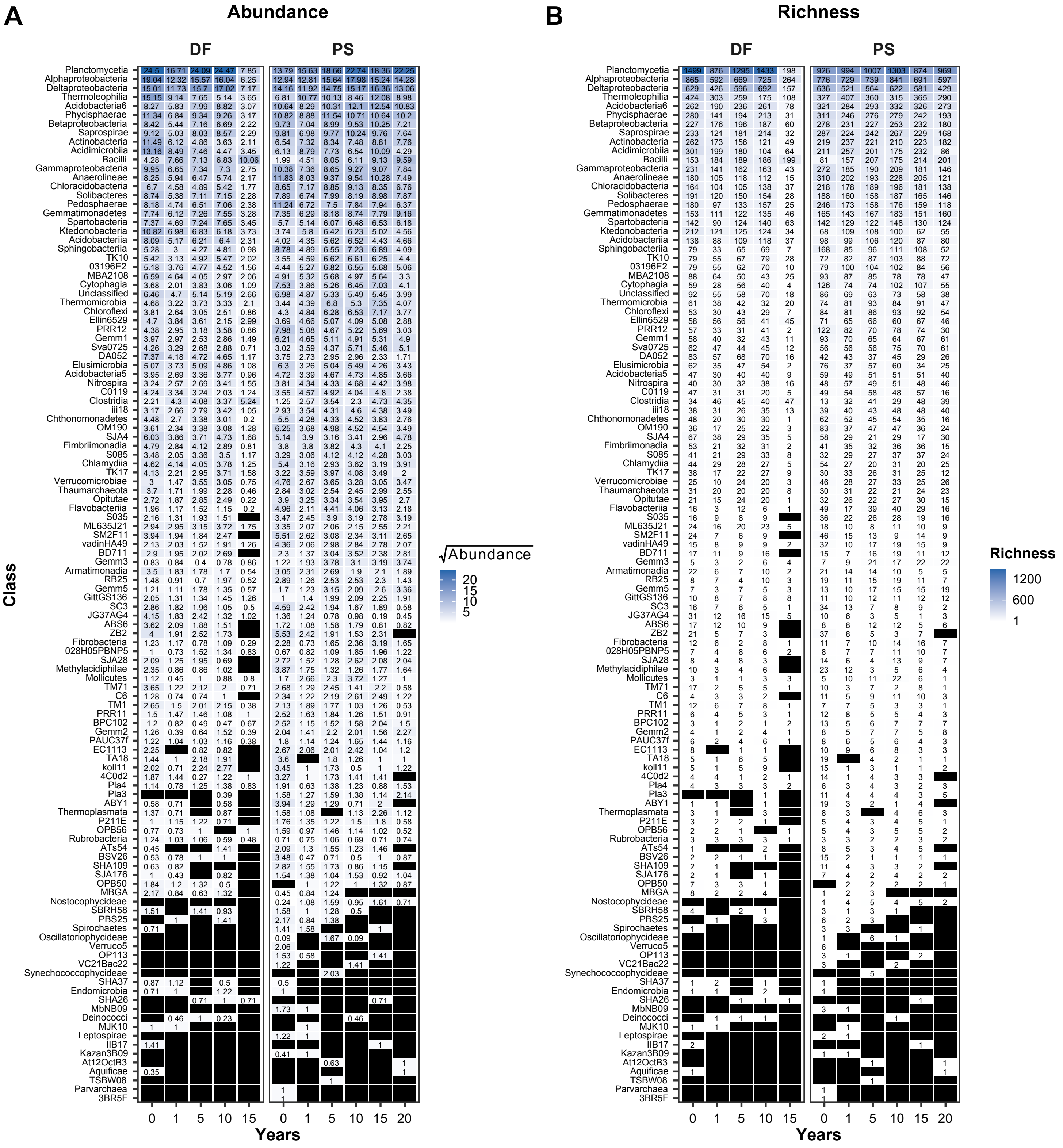

S15 Fig. Heatmap of relative abundance and OTU richness of all the prokaryotic classes. The plot shows all taxa found DF (deciduous forest) and PS (*Populus* stand) soils according the Exp1 dataset after different year of storage. Taxon relative abundance was square root transformed to improved visibility.

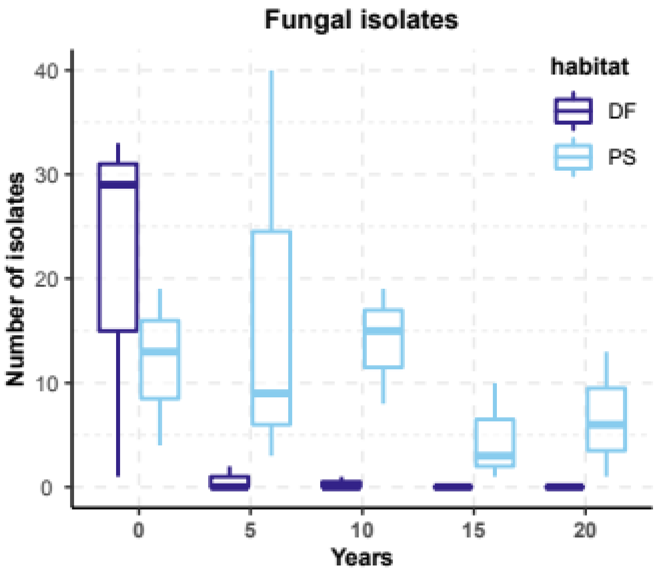

S16 Fig. Number of fungal colonies developed form DF (deciduous forest) and PS (*Populus* stand) soils. Colonies were counted 3 times over a 10 days' time period.

Isolate name	sequence ID	Length	Qurey coverage	Identity %	Genbank ID	Habitat	Year	Culture Media	Taxonomy (GenBank)	MiSeq OTU match	Read N.
PS1_2000M-1	MH027189	638	100	99	JN899355	PS	2000	MMN	*Talaromyces ohiensis*	OTU_266	883
PS1_2000M-2	MH027190	637	100	99	JN899355	PS	2000	MMN	*Talaromyces ohiensis*	OTU_266	883
PS1_1995M-3	MH027195	700	100	99	JN899355	PS	1995	MMN	*Talaromyces ohiensis*	OTU_266	883
PS2_2000M-1	MH027196	727	100	99	JN899355	PS	2000	MMN	*Talaromyces ohiensis*	OTU_266	883
PS2_1995M-1	MH027201	745	98	99	JN899355	PS	1995	MMN	*Talaromyces ohiensis*	OTU_266	883
PS2_2000P-1	MH027199	759	98	99	JN899355	PS	2000	PDA	*Talaromyces ohiensis*	OTU_266	883
PS2_2000M-2	MH027197	719	100	99	JN899395	PS	2000	MMN	*Talaromyces flavus*	OTU_270	1040
PS2_2000M-3	MH027198	708	100	99	JN899395	PS	2000	MMN	*Talaromyces flavus*	OTU_270	1040
PS2_1995M-2	MH027202	744	100	100	KX869965	PS	1995	MMN	*Epicoccum nigrum*	OTU_409	534
PS1_2000M-3	MH027191	597	100	99	KY316389	PS	2000	MMN	*Paecilomyces tenuis*	OTU_989	73
PS3_2000P-1	MH027203	677	100	98	LC317798	PS	2000	PDA	*Talaromyces trachyspermus*	OTU_989	73
PS3_1995M-1	MH027204	657	100	98	LC317798	PS	1995	MMN	*Talaromyces trachyspermus*	OTU_989	73
PS1_2000P-1	MH027192	668	100	99	KY316389	PS	2000	PDA	*Paecilomyces tenuis*	OTU_3342	54
PS1_2000P-2	MH027193	698	99	99	KX621968	PS	2000	PDA	*Paecilomyces tenuis*	OTU_3342	54
PS1_1995M-1	MH027194	698	100	99	KX621968	PS	1995	MMN	*Paecilomyces tenuis*	OTU_3342	54
PS2_2000P-2	MH027200	669	100	99	KX621968	PS	2000	PDA	*Paecilomyces tenuis*	OTU_3342	54
PS3_1995P-1	MH027205	684	100	99	KX621968	PS	1995	PDA	*Paecilomyces tenuis*	OTU_3342	54
PS3_1995P-2	MH027206	784	100	99	KX621968	PS	1995	PDA	*Paecilomyces tenuis*	OTU_3342	54
DF2_2010R-1	MH027207	616	99	99	FR839683	DF	2010	RBA	*Auxarthron umbrinum*	OTU_305	824

S2 Table. List of sequence Isolates obtained from the oldest soils which showed growing colonies. Isolates name, sequence ID, Length, Query Coverage, Identity %, Genbank ID, Habitat, Year, Culture Media (modified Melin-Norkrans or Potato Dextrose Agar), Taxonomy (GenBank), Miseq OTU match (OTU name in the Miseq data), Read N. (Number), are reported.
